# The DsbA-L paradox in protective and pathogenic stress adaptation

**DOI:** 10.1016/j.isci.2026.117449

**Published:** 2026-09-09

**Authors:** Yi Deng, Long Yan, Xianling Liu, Ting Xiao, Wen Meng

**Affiliations:** 1 Department of Oncology, The Second Xiangya Hospital of Central South University, Changsha 410011, Hunan, China; 2 National Clinical Research Center for Metabolic Diseases, The Second Xiangya Hospital of Central South University, Changsha 410011, Hunan, China; 3 Metabolic Syndrome Research Center, The Second Xiangya Hospital of Central South University, Changsha 410011, Hunan, China; 4 Department of Hepatopathy and Endocrinology, The Affiliated Children’s Hospital of Xiangya School of Medicine, Central South University (Hunan Children’s Hospital), Changsha 410007, Hunan, China

**Keywords:** DsbA-L, GSTK1, adiponectin, stress adaptation, metabolic disease

## Abstract

Disulfide-bond A oxidoreductase-like protein (DsbA-L), also known as glutathione S-transferase kappa 1 (GSTK1), regulates adiponectin assembly, organelle homeostasis, and cellular responses to metabolic stress. Evidence from cellular knockdown, constitutive and tissue-specific knockout models, and studies of metabolic, fibrotic, immune, and cancer contexts shows that its effects are strongly context dependent. DsbA-L can preserve metabolic homeostasis by supporting adiponectin multimerization, mitochondria-associated membrane integrity, redox control, mitophagy, and reticulophagy, yet it can also sustain profibrotic or immune-metabolic programs and show divergent effects across cancers. We propose that these observations are best understood by viewing DsbA-L as a regulator of stress adaptability whose output depends on cell identity, stress reversibility, downstream signaling, and adaptive reserve. This framework reconciles apparently conflicting findings and argues for tissue-specific modulation or downstream-effector targeting rather than indiscriminate systemic activation or inhibition.

## Graphical abstract

**Figure:**
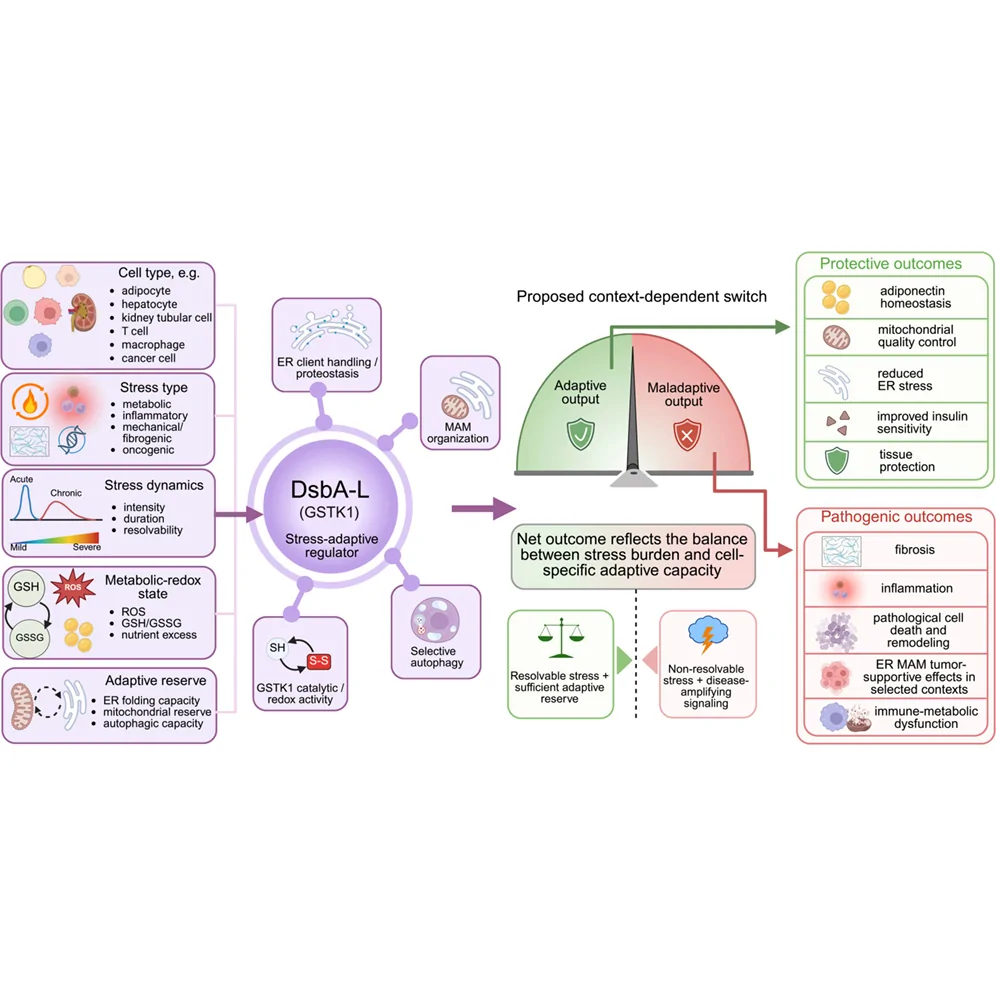

## Teaser

Biological sciences; Human metabolism; Disease in systems toxicology

## DsbA-L—From consensus to paradox

Disulfide-bond A oxidoreductase-like protein (DsbA-L), also known as glutathione S-transferase kappa 1 (GSTK1), has long been recognized as a key molecular chaperone that maintains metabolic homeostasis, primarily by facilitating the assembly of high-molecular-weight (HMW) adiponectin multimers in adipocytes.^1^^,^^2^^,^^3^^,^^4^ Adiponectin, an insulin-sensitizing and anti-inflammatory adipokine, exerts its beneficial effects predominantly in its HMW form.^5^^,^^6^^,^^7^ The expression of DsbA-L is positively regulated by PPARγ and AMPK, and is suppressed under conditions of obesity, diabetes, and metabolic dysfunction-associated steatotic liver disease (MASLD).^3^^,^^4^^,^^8^^,^^9^^,^^10^^,^^11^ Overexpression of DsbA-L in adipose tissue or liver protects mice from diet-induced obesity, insulin resistance, and hepatic steatosis, while its deficiency exacerbates these phenotypes.^10^^,^^12^^,^^13^^,^^14^ These findings have collectively established DsbA-L as a metabolic safeguard and a promising therapeutic target.^15^^,^^16^^,^^17^^,^^18^

However, a growing body of evidence challenges this simple protective narrative. Several contradictory observations have emerged that cannot be reconciled by the canonical adiponectin-centric model. First, while cellular knockdown of DsbA-L severely impairs HMW adiponectin secretion,^1^^,^^4^ global DsbA-L knockout mice unexpectedly display preserved adiponectin multimerization and secretion.^19^^,^^20^ This discrepancy between acute loss-of-function *in vitro* and constitutive ablation *in vivo* remains unexplained. Second, DsbA-L exhibits diametrically opposite roles across different pathological contexts: it protects against metabolic dysfunction in adipose tissue and liver,^10^^,^^12^^,^^13^^,^^14^ yet it promotes tissue fibrosis in the kidney and lung by activating TGF-β1/SMAD3 signaling and M2 macrophage polarization.^21^^,^^22^^,^^23^ Third, the same protein can produce cell-type-specific outcomes even within the same disease setting. For example, DsbA-L deficiency in adipocytes exacerbates obesity-induced inflammation,^24^ whereas its specific deletion in T cells improves systemic metabolism by suppressing IFN-γ production and enhancing brown adipose tissue (BAT) thermogenesis.^25^^,^^26^ These observations indicate that DsbA-L is not intrinsically protective; rather, its functional output is highly context dependent.

Several recent reviews have summarized the protective roles of DsbA-L in metabolic diseases, focusing on its effects on adiponectin multimerization, mitochondrial homeostasis, and the cGAS-STING pathway.^17^^,^^18^ While these works provide valuable foundations, they largely adopt a linear narrative in which DsbA-L deficiency is uniformly detrimental and its upregulation is universally beneficial. Consequently, the mechanistic basis of the contradictions described previously, specifically the knockout versus knockdown paradox, the protective-to-pathogenic switch, and the cell-type-specific divergence, has received little systematic attention.

We propose that these discrepancies can be reconciled by viewing DsbA-L as a context dependent regulator of cellular stress adaptation rather than as a dedicated metabolic protector. When stress can be resolved and the adaptive capacity of the cell remains sufficient, DsbA-L may support cellular recovery and tissue homeostasis. These protective effects may involve ER client handling, proteostasis, catalytic and redox activity, mitochondria-associated membranes (MAM) organization, and selective autophagy. When stress persists or cannot be resolved, these functions may produce different outcomes. They may also become harmful when they are linked to signaling pathways that amplify disease. Under these conditions, DsbA-L may contribute to fibrosis, inflammation, pathological remodeling, immune metabolic dysfunction, or tumor support in selected settings.

**Figure 1:**
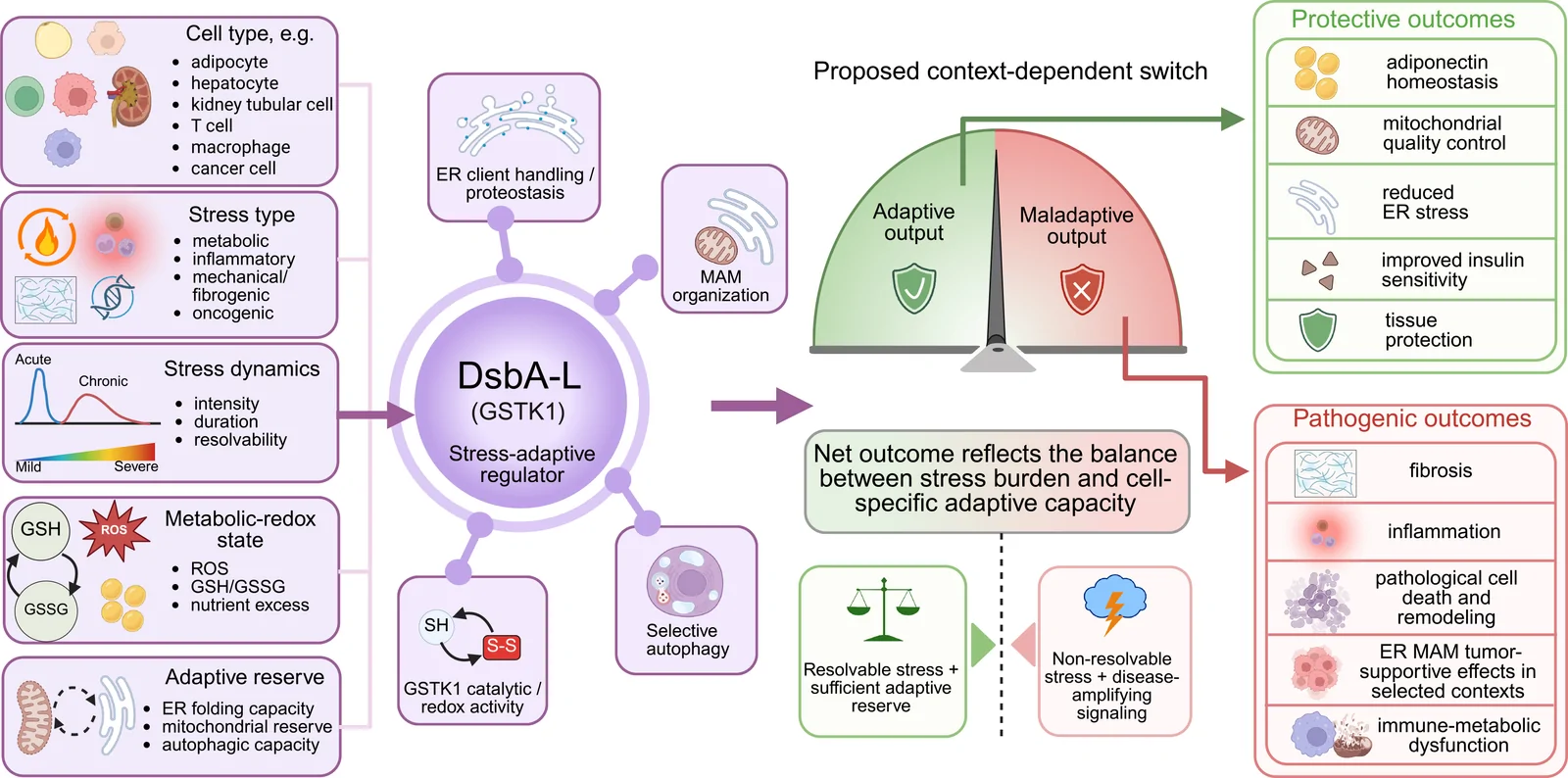
DsbA-L as a context dependent stress-adaptive regulator and proposed protective-to-pathogenic switch This schematic summarizes the framework proposed in this review. Cell identity, stress properties, metabolic-redox state, and adaptive reserve shape DsbA-L-associated ER proteostasis, MAM organization, GSTK1 catalytic/redox activity, and selective autophagy. Resolvable stress with sufficient adaptive capacity favors protective outcomes, whereas non-resolvable stress coupled to disease-amplifying signaling may promote fibrosis, inflammation, pathological remodeling, tumor-supportive effects in selected contexts, or immune-metabolic dysfunction. The proposed switch represents a context-dependent continuum rather than an established binary threshold, and the components shown are representative rather than exhaustive.

Figure 1 provides an overview of this framework. It summarizes the contextual inputs, the main functions linked to DsbA-L, and the proposed transition between adaptive and maladaptive outcomes. The model suggests that the biological effect of DsbA-L is not determined by its abundance alone. It also depends on the balance between stress burden and the adaptive capacity of the specific cell type. This proposed switch is a testable working model. It should not be interpreted as an established binary process or as a defined quantitative threshold. Before applying this framework to different tissues and diseases, the disputed role of DsbA-L in adiponectin regulation must first be reconsidered.

## The adiponectin paradox revisited: A question of essentiality

The adiponectin-centric view of DsbA-L derives primarily from cellular studies. In cultured adipocytes and heterologous expression systems, DsbA-L physically interacts with adiponectin and co-localizes with ER chaperones Ero1-Lα and ERp44.^1^^,^^3^^,^^6^ Overexpression of DsbA-L increases HMW adiponectin secretion, whereas siRNA-mediated knockdown specifically impairs multimerization without affecting adiponectin mRNA levels.^1^^,^^4^ The N-terminal Ser-Pro-Tyr-Ser motif is critical for this function, as its mutation abrogates trimer-to-multimer conversion.^1^ DsbA-L expression is positively regulated by PPARγ (and enhanced by thiazolidinediones) and suppressed by pro-inflammatory cytokines such as TNF-α.^8^^,^^9^^,^^24^^,^^27^ These observations have led to the prevailing view that DsbA-L is indispensable for bioactive adiponectin production.

However, this conclusion is challenged by evidence from constitutive whole-body DsbA-L knockout mice.^19^^,^^20^ Although structural studies have characterized DsbA-L at the molecular level,^19^ constitutive Gstk1 deficiency does not significantly impair adiponectin multimerization *in vivo*.^20^ DsbA-L knockout mice show no detectable defect in adiponectin oligomer distribution.^20^ This discrepancy between acute cellular knockdown and chronic genetic ablation is striking: if DsbA-L is truly essential for adiponectin assembly, why is multimerization preserved after constitutive loss?

Developmental compensation may explain the preservation of adiponectin multimerization, but such compensation is unlikely to restore every function of DsbA-L. Lifelong loss of DsbA-L may induce parallel ER folding and protein quality control pathways.^20^^,^^28^ These pathways may preserve adiponectin assembly in the constitutive knockout model.^19^^,^^20^ This possibility should be described as selective compensation rather than complete functional restoration.

Other functions of DsbA-L may remain only partly compensated or may not be compensated at all. These functions include mitochondrial regulation, peroxisomal redox control, MAM organization, and cell type-specific signaling.^10^^,^^14^^,^^29^^,^^30^^,^^31^^,^^32^ A separate study of Gstk1 knockout mice found glomerular basement membrane abnormalities, microalbuminuria, and podocyte foot process effacement.^33^ These abnormalities occurred without evidence of generalized oxidative stress. This finding shows that preserved adiponectin multimerization can coexist with tissue pathology.

MAM dysfunction is therefore a plausible example of an uncompensated function, but it has not been demonstrated in the constitutive whole body knockout model. MAM integrity was not measured in the study that examined adiponectin multimerization. Evidence from diabetic kidney models shows that DsbA-L supports MAM integrity and mitophagy.^29^^,^^30^ These findings establish MAM regulation as an important function of DsbA-L. They do not prove that this function remains defective in the constitutive knockout mouse.

The constitutive whole body knockout model should also be distinguished from tissue specific loss of function models. Fat specific DsbA-L deletion worsens obesity associated inflammation and insulin resistance.^14^ Hepatic DsbA-L deletion aggravates diet induced steatosis and insulin resistance.^10^ These findings demonstrate that DsbA-L has important functions in adult metabolic tissues under stress. They do not show that the constitutive whole body knockout has an exacerbated basal systemic metabolic phenotype. Differences in tissue, timing of deletion, dietary challenge, and developmental adaptation may account for the distinct outcomes.

This paradox suggests that the field’s heavy reliance on cellular knockdown models may have overestimated the essentiality of DsbA-L for adiponectin multimerization *in vivo*. It also raises a more fundamental question: does DsbA-L primarily exist to chaperone adiponectin, or does adiponectin merely serve as a convenient reporter of DsbA-L’s broader chaperone activity? Key knowledge gaps remain. An adipocyte specific inducible DsbA-L knockout is needed to determine whether acute deletion in mature adipocytes reproduces the adiponectin defect observed in cultured cells. The same model should also be used to compare adiponectin multimerization with mitochondrial function, MAM organization, redox balance, and autophagic flux. This approach would test whether compensation preserves one functional output while leaving other DsbA-L dependent processes impaired. The full interactome of DsbA-L, its binding partners in different cell types and under different stress conditions, remains completely unknown. And whether post-translational modifications or metabolic cues dynamically regulate DsbA-L’s client specificity has not been explored.

The adiponectin paradox thus forces a reconceptualization: DsbA-L operates not as a dedicated adiponectin chaperone, but as a broad-spectrum, context-sensitive ER quality control hub with adiponectin representing merely one stress-responsive client among potentially many. If this principle holds, DsbA-L should exhibit functional plasticity, perhaps even functional inversion, across different tissues and pathological milieus. The following section systematically interrogates this prediction, documenting how DsbA-L transitions from metabolic protector to pathological driver depending on cellular context.

## Context-dependent outcomes: From protection to pathogenesis

The functional output of DsbA-L is exquisitely context dependent. In classical metabolic tissues, it enhances energy homeostasis; in immune cells, it modulates inflammatory tone; yet in chronic fibrotic or oncogenic environments, the identical stress-adaptive machinery becomes subverted to drive pathological remodeling. This section dissects context dependence across three distinct layers: tissue-specific functional divergence, immune-metabolic functional inversion, and the transition from protective adaptation to maladaptive pathology in fibrosis and cancer.

In classical metabolic tissues such as adipose tissue and liver, DsbA-L consistently exerts protective effects. Adipose-specific overexpression of DsbA-L enhances HMW adiponectin secretion, reduces diet-induced obesity, and improves insulin sensitivity.^12^^,^^13^ Conversely, fat-specific DsbA-L knockout exacerbates obesity-related inflammation and insulin resistance by promoting mitochondrial DNA (mtDNA) release and activating the cGAS-cGAMP-STING pathway.^14^ In the liver, hepatocyte-specific DsbA-L deficiency aggravates high-fat diet-induced steatosis and insulin resistance, while hepatic overexpression ameliorates these phenotypes.^10^ These observations establish DsbA-L as a protective node in adipocytes and hepatocytes. However, even within metabolic tissues, the response is not monolithic. In the kidney, DsbA-L protects against diabetic nephropathy by maintaining MAM integrity and activating mitophagy.^29^^,^^30^^,^^34^^,^^35^ Yet, under ureteral obstruction, a model of chronic mechanical and inflammatory stress, DsbA-L promotes tubulointerstitial fibrosis by interacting with VDAC1 and activating apoptotic pathways.^22^^,^^36^^,^^37^ Thus, the same protein can be protective or pathogenic in the same organ depending on the nature of the insult (metabolic vs. mechanical/fibrotic).

Perhaps the most striking example of context dependence comes from studies of DsbA-L in immune cells. While DsbA-L deficiency in adipocytes worsens metabolic dysfunction,^14^ its specific deletion in T cells produces the opposite effect. T cell-specific DsbA-L knockout protects mice from high-fat diet-induced obesity and insulin resistance.^25^^,^^26^ Mechanistically, DsbA-L deficiency in T cells suppresses IFN-γ production by reducing phosphodiesterase-4D expression, which in turn enhances BAT thermogenesis via protein kinase A activation.^26^ This finding reveals a functional inversion: in adipocytes, DsbA-L promotes metabolic health; in T cells, it contributes to metabolic dysfunction by sustaining an inflammatory tone. The broader implication is that DsbA-L’s role in the immune system may fundamentally differ from its role in parenchymal metabolic tissues. This immune-metabolic axis has been largely overlooked by previous reviews, yet it carries profound implications for therapeutic targeting: systemic DsbA-L activation could inadvertently suppress beneficial thermogenic programs by modulating T cell function.

**Figure 2:**
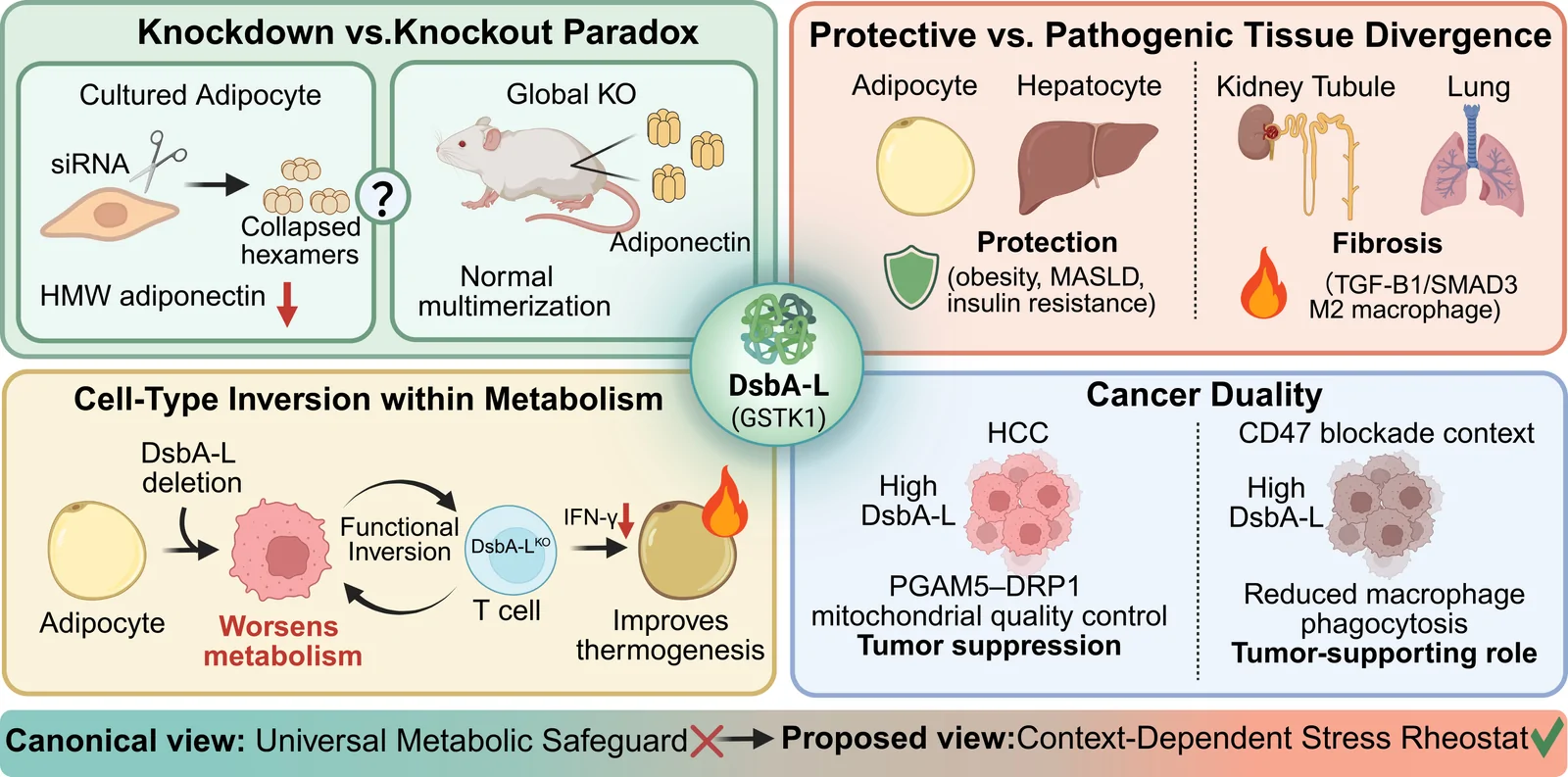
The DsbA-L paradox across genetic tissue and disease contexts This figure summarizes observations that challenge a universal protective model of DsbA-L. Acute knockdown in cultured adipocytes impairs HMW adiponectin multimerization, whereas constitutive global knockout preserves relatively normal multimerization. DsbA-L is protective in adipocytes and hepatocytes but can promote fibrotic programs in kidney and lung. Its loss worsens metabolic dysfunction in adipocytes yet improves thermogenesis in T cell-specific knockout models through reduced IFN-γ signaling. In cancer, GSTK1/DsbA-L is tumor suppressive in HCC, whereas in a CD47-blockade context GSTK1 can limit macrophage phagocytosis, illustrating cancer-context-dependent duality.

The stress-adaptive functions of DsbA-L, maintaining ER proteostasis, mitigating oxidative stress, and supporting mitochondrial integrity, are beneficial under acute or mild stress. However, under chronic or aberrant stress, the same adaptive mechanisms can become maladaptive, driving pathological tissue remodeling and tumor progression. In renal fibrosis, DsbA-L promotes extracellular matrix accumulation by activating TGF-β1/SMAD3 signaling in tubular epithelial cells and fibroblasts.^21^^,^^22^ Mechanistically, DsbA-L interacts with HSP90 in mitochondria to enhance SMAD3 phosphorylation and CTGF expression.^22^ In pulmonary fibrosis, DsbA-L drives disease progression by stimulating AKT1 and NLRP3 inflammasome activation, which polarizes macrophages toward a pro-fibrotic M2 phenotype.^2^^,^^23^ In cancer, the role of DsbA-L is similarly dualistic. Low DsbA-L expression has been associated with poor prognosis in head and neck squamous cell carcinoma,^38^^,^^39^ suggesting a potential tumor-suppressive role. Conversely, DsbA-L knockout promotes hepatocellular carcinoma progression by regulating L-carnitine metabolism and mitochondrial quality control via the PGAM5/DRP1 complex.^40^ In breast cancer, DsbA-L has been identified as a prognostic biomarker associated with immune infiltration.^41^ Moreover, dual pharmacological blockade of DsbA-L and CD47 synergistically enhances macrophage-mediated phagocytosis of cancer cells, pointing to a pro-survival role of DsbA-L in the tumor microenvironment.^42^ Taken together, current evidence supports cancer-context-dependent functions of DsbA-L/GSTK1 rather than a uniformly oncogenic or tumor-suppressive role. Its effects appear to depend on tumor type, mitochondrial programs, and interactions with the immune microenvironment (Figure 2).

Collectively, these findings establish that DsbA-L functions fundamentally as a cellular stress adaptability rheostat, a regulatory node whose functional plasticity is not an experimental nuisance but an intrinsic design feature. These observations also show that tissue identity alone cannot explain the functional outcome of DsbA-L. The outcome depends on whether the stress can be resolved, which signaling pathway becomes dominant, and whether the affected cell retains sufficient adaptive capacity.^14^^,^^22^^,^^23^^,^^26^ This characterization raises a critical mechanistic question: what molecular architecture enables DsbA-L to sense diverse contextual inputs and translate them into divergent biological outputs? The following section reveals how DsbA-L integrates organelle communication, metabolic signaling, and proteostatic control to achieve context-responsive stress adaptation.

## Mechanistic basis of context-dependency: DsbA-L as a stress integration node

Understanding the context dependence of DsbA-L requires viewing it as a stress integration node with several molecular functions. DsbA-L is identical to GSTK1, a kappa class glutathione transferase with measurable catalytic activity.^31^ It also supports adiponectin multimerization and ER proteostasis through protein interactions and subcellular localization.^1^^,^^3^^,^^43^ How these catalytic and chaperone related functions are coordinated remains unresolved. Their relative contributions are likely to vary across cell types, cellular compartments, and stress conditions.

Structural and kinetic studies identify Ser16 as an important residue for GSH dependent catalysis by human GSTK1.^31^ Mutation of the same residue also reduces the interaction between DsbA-L and adiponectin and impairs higher order adiponectin multimerization.^1^ This overlap suggests that the enzymatic and chaperone related functions may share a structural basis. However, it does not prove that GSTK1 catalysis directly drives adiponectin folding or assembly. Mutation of Ser16 may affect catalytic activity, local structure, and protein binding at the same time.

The enzymatic and chaperone related functions may interact in several ways. GSTK1 activity may limit oxidative and electrophilic damage and thereby preserve conditions that support protein handling and organelle quality control. Studies in cultured cells show that loss of GSTK1 delays the recovery of peroxisomal redox balance after oxidative stress.^44^ Functional separation by subcellular location is also possible. ER localization is important for the adiponectin related function, while peroxisomal localization may favor catalytic and redox regulation.^43^ Direct competition is less well supported. Severe oxidative stress may increase the demand for GSH dependent detoxification, but current studies have not shown that GST substrates displace adiponectin or that increased catalytic activity suppresses the chaperone related function. Competition should therefore be considered a testable hypothesis rather than an established mechanism.

Subcellular localization provides another basis for the context dependence of DsbA-L. DsbA-L has been detected in the ER, mitochondria, peroxisomes, and MAMs.^20^^,^^29^^,^^32^ This subcellular distribution enables DsbA-L to coordinate inter-organelle communication, which is increasingly recognized as a key determinant of cellular fate under stress. The MAM, a specialized contact site between the ER and mitochondria, has emerged as a central platform where DsbA-L exerts its regulatory functions. MAMs regulate calcium transfer, lipid metabolism, mitochondrial dynamics, and autophagosome formation.^16^^,^^45^ DsbA-L localizes to MAMs and is essential for maintaining their structural integrity.^29^^,^^34^ In renal tubular cells, DsbA-L deficiency disrupts MAM integrity, leading to impaired calcium homeostasis, reduced mitophagy, and exacerbated mitochondrial fragmentation.^30^^,^^34^^,^^35^ Conversely, DsbA-L overexpression preserves MAM integrity and protects against high glucose-induced tubular damage.^29^ The interaction between DsbA-L and VDAC1, a mitochondrial outer membrane protein that forms part of the IP3R-GRP75-VDAC1 calcium transfer complex, may contribute to MAM stabilization.^24^^,^^36^^,^^37^ The functional output of DsbA-L is thus dictated by the organellar context. In metabolic tissues where MAM integrity is critical for insulin sensitivity and lipid homeostasis, DsbA-L’s MAM-stabilizing function translates into protection. However, in pathological settings where sustained ER-mitochondria coupling promotes excessive calcium transfer and mitochondrial apoptosis (e.g., in fibrosis or ischemia-reperfusion injury), the same MAM interaction may become detrimental.^36^^,^^37^ This duality underscores the principle that organelle coupling does not have an intrinsic valence; its effect depends on the broader stress landscape.

Second, beyond its structural role in organelle communication, DsbA-L is directly regulated by metabolic intermediates that link cellular energy status to protein folding and secretion. A recently discovered mechanism involves the inositol pyrophosphate 5-InsP7, synthesized by hexakisphosphate kinase 1 (IP6K1).^46^ Under basal conditions, 5-InsP7 stabilizes the interaction between the adiponectin-retention chaperone ERp44 and the DsbA-L/Ero1-Lα complex, thereby promoting thiol-mediated retention and degradation of adiponectin within the ER. Pharmacological or genetic inhibition of IP6K1 lowers 5-InsP7 levels, disrupts this complex, and enhances the secretion of bioactive HMW adiponectin, leading to cardioprotective effects.^46^^,^^47^ This discovery is significant for two reasons. First, it establishes a direct link between metabolic flux (via inositol phosphate metabolism) and DsbA-L-dependent protein secretion. Second, it provides a proof-of-principle that DsbA-L activity can be pharmacologically modulated by targeting its upstream regulators rather than the protein itself. Notably, to date, this is the only known post-translational mechanism that directly modulates DsbA-L function in response to metabolic cues. Whether other metabolites such as NAD+, acetyl-CoA, the GSH/GSSG ratio, or reactive oxygen species similarly regulate DsbA-L activity, localization, or client specificity remains entirely unexplored. The implication for context dependence is clear: the same DsbA-L molecule may exhibit different activities depending on the metabolic state of the cell. In conditions of high 5-InsP7 (e.g., nutrient excess), DsbA-L is biased toward adiponectin retention; when 5-InsP7 is low (e.g., energy restriction or IP6K1 inhibition), DsbA-L promotes adiponectin secretion.

Third, the ultimate outcome of DsbA-L activity, whether protective or pathogenic, is also shaped by its integration with autophagy and protein quality control pathways. Emerging evidence indicates that DsbA-L participates in selective autophagic processes, particularly reticulophagy (ER-phagy) and mitophagy. In diabetic nephropathy, DsbA-L-mediated reticulophagy attenuates tubular injury by clearing damaged ER fragments and reducing ER stress-induced apoptosis.^48^ This mechanism involves the reticulophagy receptor RETREG1/FAM134B and appears to be specific to ER stress conditions. Similarly, DsbA-L promotes mitophagy by maintaining MAM integrity through a HELZ2-PPARα-MFN2-associated mechanism.^30^^,^^35^ In the liver, DsbA-L suppresses hepatocellular carcinoma progression by enhancing mitochondrial quality control through the PGAM5/DRP1 complex, which regulates mitophagy and mitochondrial dynamics.^40^ These findings position DsbA-L as a rheostat of proteostatic health. When cellular repair capacity remains sufficient, DsbA-L associated autophagy may remove damaged organelles and help restore homeostasis.^29^^,^^30^^,^^48^ When stress cannot be resolved, these protective effects may become insufficient, while other DsbA-L associated pathways may support pathological remodeling.^22^^,^^23^ The outcome therefore depends on more than stress duration alone.

The transition from a protective role to a pathogenic role cannot be explained by a simple distinction between acute and chronic stress. Current studies have not identified a single threshold for DsbA-L expression, stress duration, MAM function, or autophagic flux. A more useful boundary is whether the stress can still be resolved by the adaptive capacity of the cell. When repair systems remain effective, DsbA-L can support recovery and restore homeostasis. When stress persists and repair capacity becomes insufficient, the same functions may maintain or amplify a pathological state.

Several factors shape this boundary. The first is the nature and reversibility of the stress. Metabolic stress caused by high glucose may remain partly reversible when mitochondrial quality control and autophagy are preserved.^29^^,^^30^^,^^48^ Persistent obstruction and fibrotic signaling are less likely to be resolved and may continuously activate tissue remodeling pathways.^22^ The second factor is cell identity. Preservation of mitochondrial and ER function can support metabolic homeostasis in adipocytes and hepatocytes.^10^^,^^13^^,^^14^ In T cells and macrophages, however, DsbA-L can support signaling programs that worsen systemic metabolism or promote fibrosis.^23^^,^^26^ The third factor is the dominant downstream pathway. DsbA-L is more likely to be protective when MAM organization, mitophagy, and reticulophagy promote the clearance of damaged organelles.^29^^,^^30^^,^^48^ It is more likely to be pathogenic when HSP90, SMAD3, AKT1, NLRP3, or related disease amplifying pathways become dominant.^22^^,^^23^

The fourth factor is adaptive reserve. This reserve includes mitochondrial capacity, ER folding capacity, redox buffering, and autophagic flux. DsbA-L is more likely to remain protective when these systems can remove damaged proteins and organelles and restore cellular function.^29^^,^^30^^,^^48^ It is more likely to become maladaptive when these systems are insufficient or when other DsbA-L associated pathways sustain fibrosis or inflammation.^22^^,^^23^ The boundary is therefore not defined by stress duration alone. It is defined by the interaction among stress reversibility, cell identity, downstream signaling, and adaptive reserve. These conditions form a testable framework rather than a fixed switch point.

**Figure 3:**
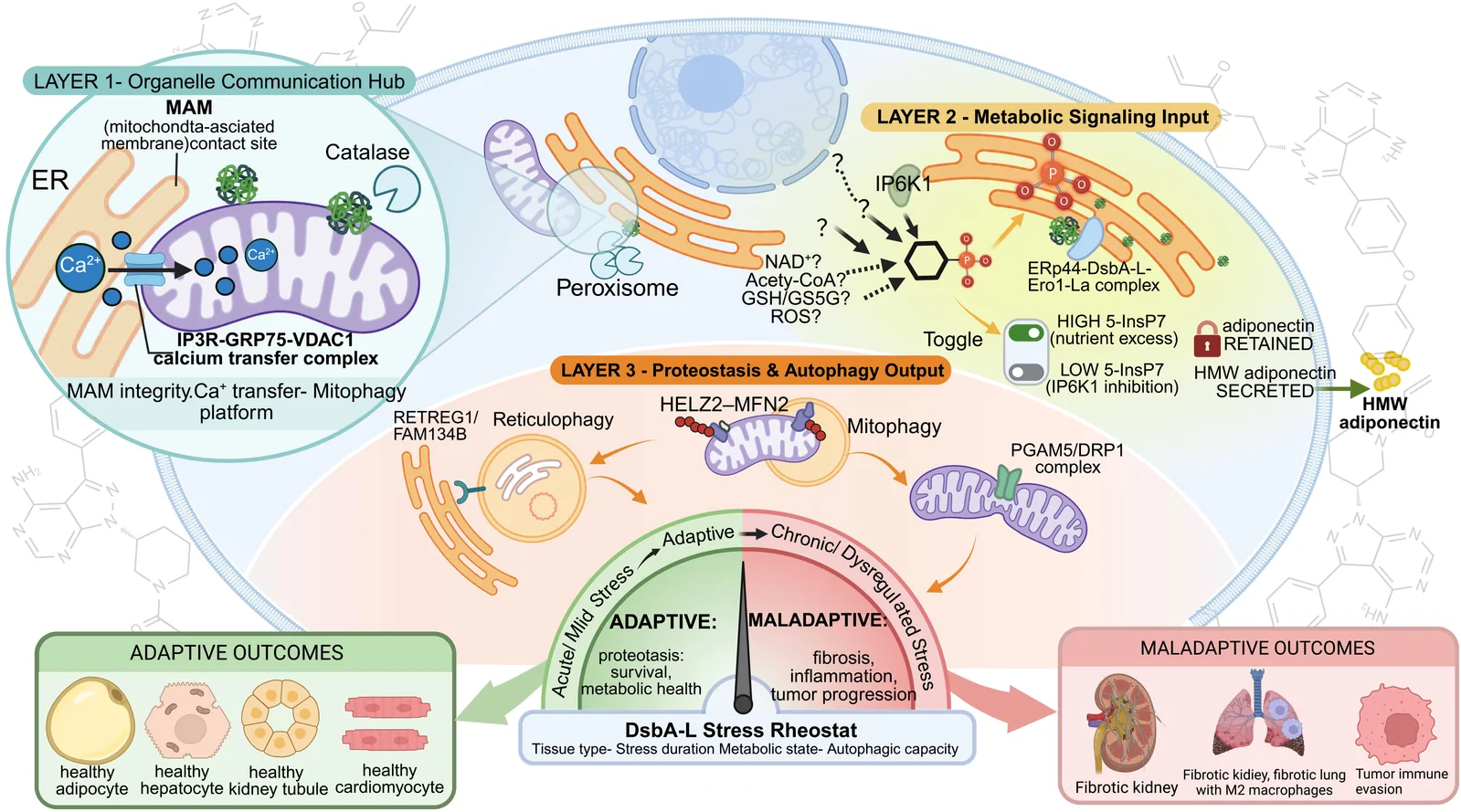
DsbA-L as a stress rheostat integrating organelle communication metabolic input and proteostatic output This model illustrates how DsbA-L integrates cellular stress responses through MAM-associated organelle communication, IP6K1/5-InsP7-dependent adiponectin secretory control, and selective autophagy. MAM integrity supports calcium homeostasis and mitophagy, while reticulophagy and mitochondrial quality-control pathways provide additional proteostatic outputs. These processes favor recovery when stress remains resolvable but may coexist with fibrosis, inflammation, pathological remodeling, immune-metabolic dysfunction, or tumor-supportive effects when adaptive capacity is exceeded and disease-amplifying pathways dominate.

These mechanisms are functionally connected. DsbA-L may link GSTK1 catalytic and redox activity with ER client handling, organelle communication, metabolic regulation, and selective autophagy. The contribution of each function is likely to vary across cell types and stress conditions. Their causal relationships remain incompletely defined.^29^^,^^30^^,^^47^^,^^48^ Its net effect depends on cell identity, stress reversibility, the dominant downstream pathway, and the remaining adaptive reserve of the cell. As summarized in Figures 1 and 3, DsbA-L is more likely to support recovery when stress can be resolved. It is more likely to sustain pathological remodeling when stress persists and disease amplifying pathways become dominant.^22^^,^^23^ We propose that DsbA-L acts as a stress adaptability rheostat. Under conditions where stress is acute and resolvable, DsbA-L enhances cellular fitness and promotes recovery; under chronic, unresolvable, or dysregulated stress, its adaptive functions become maladaptive, contributing to fibrosis, inflammation, or tumor progression. This stress adaptability rheostat framework unifies the apparently contradictory observations cataloged in preceding sections, yet simultaneously exposes a fundamental therapeutic paradox. If DsbA-L’s functional essence is context dependent, then systemic activation or inhibition must yield unpredictable consequences, beneficial in metabolic tissues, potentially deleterious in fibrotic or neoplastic contexts. This contextual duality renders DsbA-L a therapeutically elusive target. The following section systematically analyzes the therapeutic challenges arising from this molecular architecture, and explores potential strategies for precision intervention.

## Therapeutic implications, challenges, and precision strategies

**Figure 4:**
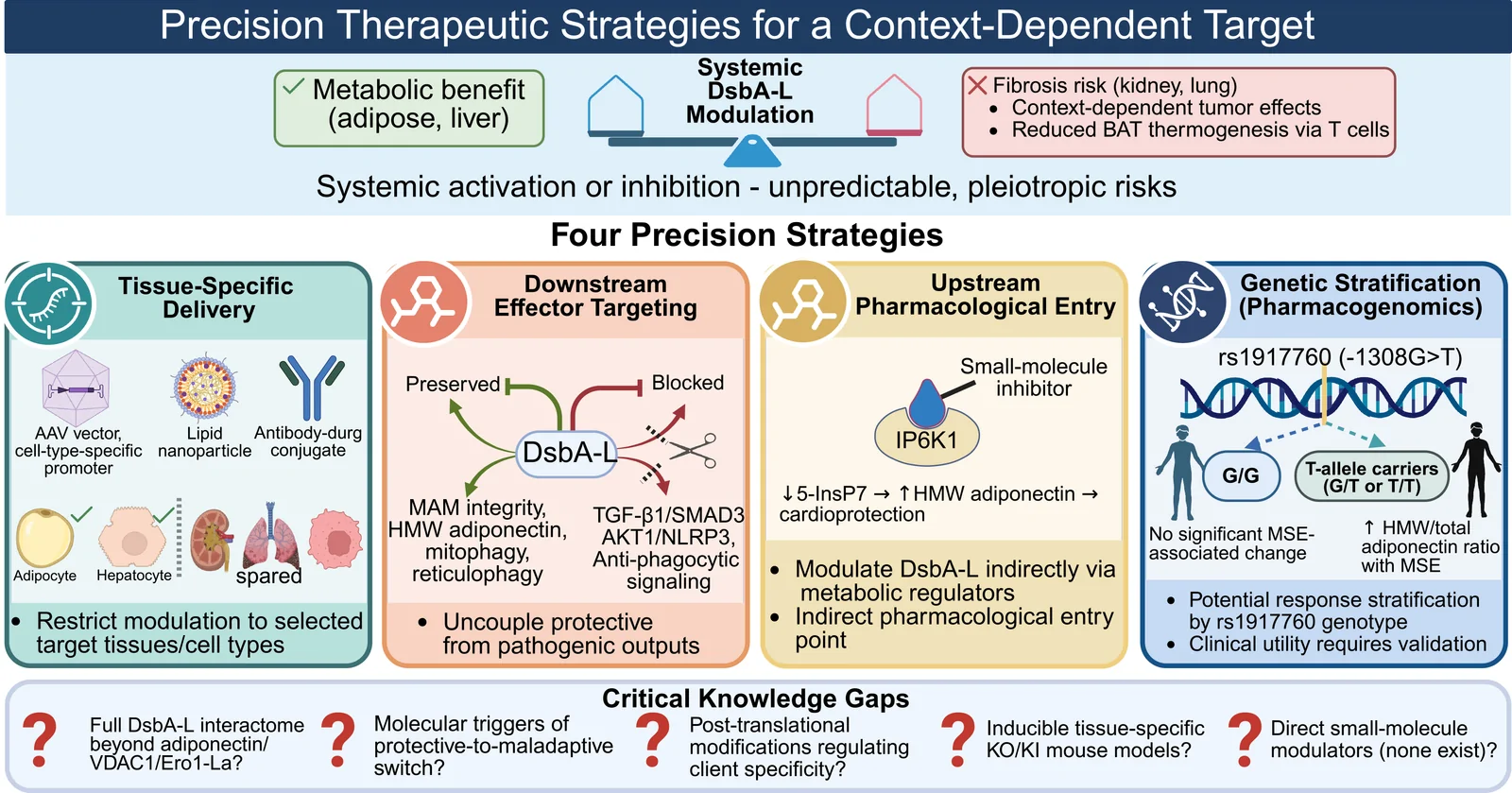
Precision therapeutic strategies for targeting the context-dependent DsbA-L axis Because DsbA-L can exert opposing effects across tissues and disease contexts, systemic activation or inhibition may produce unpredictable outcomes. Potential strategies include tissue- or cell-specific delivery, downstream-effector targeting to separate protective from pathogenic functions, and indirect modulation through upstream regulators such as IP6K1. Genetic stratification may provide an additional approach: the MSE-associated increase in the HMW/total adiponectin ratio was more pronounced in rs1917760 (−1308G>T) T-allele carriers than in G/G carriers. These pharmacogenomic observations require further validation. Key barriers include incomplete mapping of DsbA-L-associated interactions, the molecular triggers of functional switching, client specificity, and the lack of established direct selective modulators.

Conventional therapeutic targeting of DsbA-L assumes unidirectional, universally beneficial functions,^15^^,^^16^^,^^17^^,^^18^ but the context dependence revealed herein fundamentally invalidates this premise (Figure 4). Systemic DsbA-L activation, while potentially ameliorating metabolic disease, may inadvertently exacerbate renal or pulmonary fibrosis and could produce divergent effects in cancer; conversely, systemic inhibition might suppress pathological remodeling at the cost of metabolic deterioration. This section dissects these obstacles rooted in the stress adaptability rheostat nature of DsbA-L, and outlines a path toward precision therapeutic strategies.

A primary obstacle is the lack of direct pharmacological modulators. No specific, direct-acting small-molecule agonist or antagonist of DsbA-L currently exists. All reported compounds that upregulate DsbA-L expression, including resveratrol, L-cysteine, manganese, melinjo seed extract, and various herbal extracts, are pleiotropic and exert multiple effects on cellular metabolism.^4^^,^^7^^,^^45^^,^^49^^,^^50^^,^^51^^,^^52^ Whether their metabolic benefits are causally attributable to DsbA-L induction remains unproven. The IP6K1/5-InsP7 axis provides a proof-of-concept indirect pharmacological entry point for modulating DsbA-L-associated secretory control. Pharmacological inhibition of IP6K1 reduces 5-InsP7, increases circulating HMW adiponectin, and confers cardioprotection in experimental models.^46^^,^^47^

Even if specific modulators become available, the risks of systemic targeting pose a major safety hurdle. Systemic activation, while potentially beneficial in adipose tissue and liver,^10^^,^^12^^,^^13^ could inadvertently exacerbate fibrosis in kidney and lung^21^^,^^22^^,^^23^ or produce divergent effects across cancer contexts.^40^^,^^41^ Conversely, systemic inhibition might improve fibrotic diseases but could worsen metabolic dysfunction. The immune-metabolic inversion further complicates systemic targeting: enhancing DsbA-L activity in T cells may suppress BAT thermogenesis, whereas T cell-specific inhibition could have the opposite metabolic effect; its broader immunological consequences remain unclear.^26^ Indiscriminate systemic modulation is therefore unlikely to be safe or effective.

Genetic and expression heterogeneity adds another layer of complexity. GSTK1/DsbA-L shows tissue- and regulator-dependent expression, while genetic variation in DsbA-L has been linked to metabolic phenotypes and genotype-dependent responses to metabolic interventions.^53^^,^^54^^,^^55^^,^^56^ In particular, the rs1917760 (−1308G>T) polymorphism has been associated with body-weight-related phenotypes and NAFLD risk,^55^ and the response of HMW/total adiponectin to melinjo seed extract appears more pronounced in carriers of the G/T or T/T genotypes.^56^ The functional basis of these genotype-dependent associations remains incompletely resolved, and whether rs1917760 directly influences DsbA-L expression across relevant tissues requires further investigation. These observations nevertheless raise the possibility that rs1917760 could serve as a response-stratification marker for selected DsbA-L-related interventions. Future studies should determine whether genotype is associated with tissue-specific DsbA-L expression and whether it reproducibly predicts therapeutic response in independent cohorts. Until such validation is available, the clinical utility of rs1917760 remains uncertain.

Finally, experimental model limitations hamper progress. Most mechanistic studies rely on global or tissue-specific constitutive knockout mice, which may have developed compensatory adaptations that mask the true effects of acute DsbA-L loss.^19^^,^^20^ The discrepancy between cellular knockdown and global knockout (Section 2) underscores this concern. Inducible, tissue-specific knockout models (e.g., using CreERT2 systems) are urgently needed, and no knock-in models expressing a constitutively active or dominant-negative DsbA-L mutant exist.

**Figure 5:**
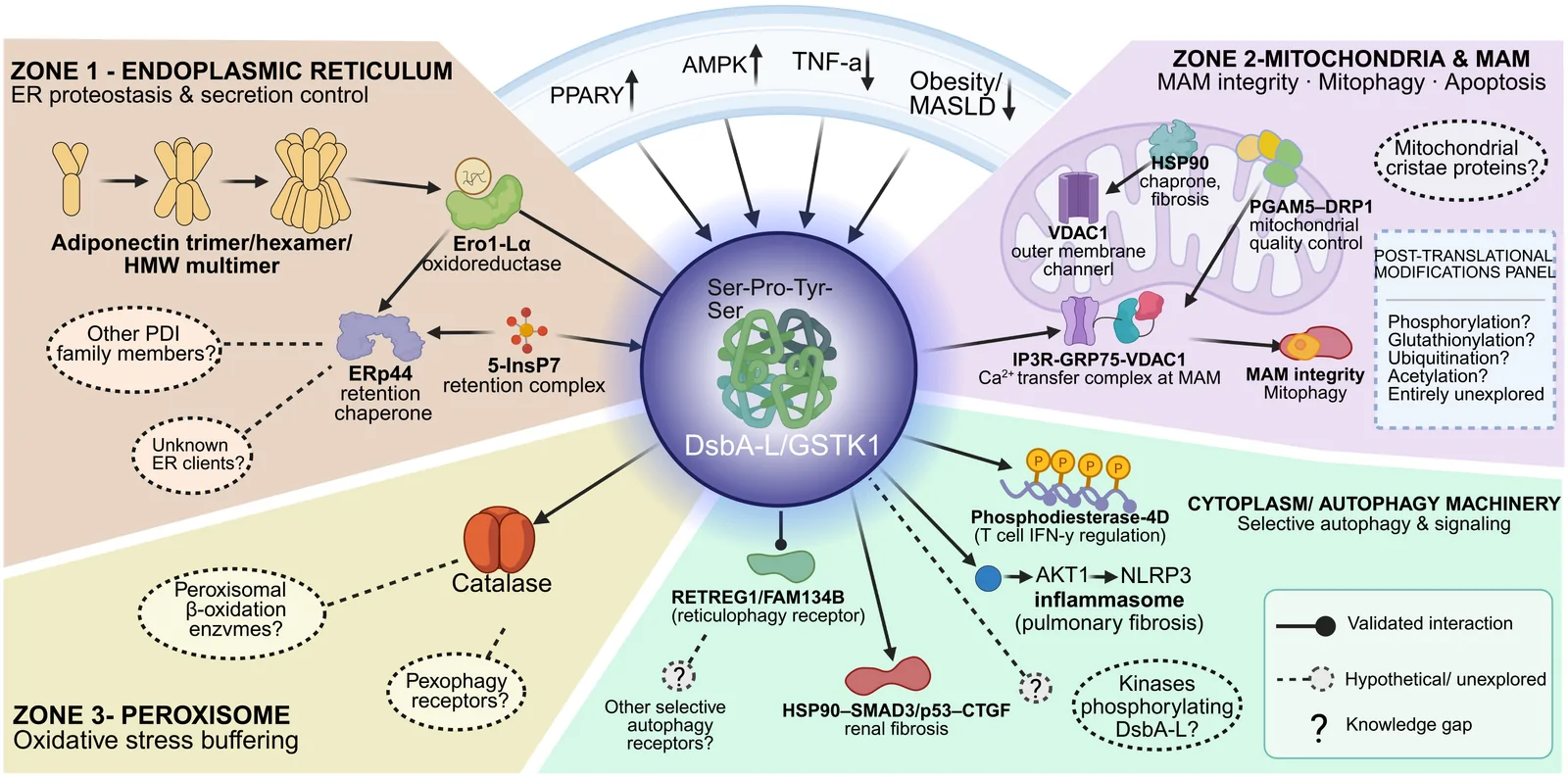
The DsbA-L functional interaction landscape and unresolved mechanistic frontiers This schematic summarizes experimentally supported and proposed DsbA-L-associated relationships across major subcellular compartments. In the ER, DsbA-L participates in adiponectin multimerization and secretory control through Ero1-Lα-, ERp44-, and 5-InsP7-related mechanisms. At mitochondria and MAMs, it is linked to VDAC1-dependent apoptosis, MAM integrity and mitophagy, HSP90-associated fibrotic signaling, and PGAM5-DRP1-mediated mitochondrial quality control. DsbA-L/GSTK1 is also linked to RETREG1/FAM134B-dependent reticulophagy, PDE4D- and AKT1/NLRP3-related signaling, and catalase-associated peroxisomal redox control. Solid lines indicate experimentally supported physical or regulatory relationships, whereas dashed lines denote hypothetical or unresolved associations. Question-mark nodes indicate major knowledge gaps.

To move beyond these obstacles, the field must address several fundamental questions. What determines tissue- and cell-type-specific function? Systematic mapping of DsbA-L’s interaction partners across relevant cell types using proximity labeling is essential. How does DsbA-L switch from protective to maladaptive? Inducible *in vivo* models and unbiased phosphoproteomics are needed to identify the molecular triggers of this transition. What is the full interactome of DsbA-L? Beyond adiponectin, VDAC1, and Ero1-Lα, the complete repertoire of client proteins remains unknown (Figure 5). How is DsbA-L regulated by metabolic signals and post-translational modifications? The IP6K1/5-InsP7 axis provides a defined example of metabolic regulation of DsbA-L-associated secretory control; whether additional metabolites or post-translational modifications regulate DsbA-L activity, localization, or client specificity remains unclear.

Separation of function models are also needed to distinguish GSTK1 catalytic activity from the chaperone related function of DsbA-L. The S16A mutant alone is not sufficient because it affects both catalytic activity and adiponectin interaction.^1^^,^^31^ Future studies should compare wild-type DsbA-L with mutants that selectively alter the catalytic pocket, ER localization, or partner binding. GST activity, redox balance, adiponectin multimerization, MAM function, and autophagic flux should be measured in the same experimental system.

Can DsbA-L functions be selectively modulated? The ultimate goal is to uncouple beneficial functions (MAM maintenance, HMW adiponectin secretion, and mitophagy) from detrimental ones (TGF-β/SMAD3 activation, cancer cell survival). Addressing these questions will require a concerted effort combining genetic, proteomic, metabolomic, and pharmacological approaches. In parallel, the development of tissue-specific delivery systems, such as adeno-associated virus vectors with cell-type-specific promoters, nanoparticles, or antibody-drug conjugates, will be essential to achieve precision targeting.

In summary, therapeutic development for DsbA-L faces an intrinsic obstacle: we cannot selectively activate its protective functions in metabolic tissues without concurrently risking deleterious outcomes in fibrotic, neoplastic, or immunological contexts. Until tissue-specific delivery systems mature, or until downstream effectors that discriminate protective from pathogenic DsbA-L functions are identified, DsbA-L remains a biologically compelling yet pharmaceutically inaccessible target. This predicament stems not from technical limitations alone, but from the fundamental molecular identity of DsbA-L as a context-dependent stress adaptability regulator rather than a dedicated metabolic safeguard.

## Conclusion

DsbA-L is not a universal metabolic safeguard. It is a context dependent regulator of cellular stress adaptability, a rheostat tuned by tissue type, stress nature, and duration. This framework reconciles the field’s central contradictions: protective in metabolic tissues, pathogenic in fibrotic settings, context dependent in cancer, and functionally divergent across cell types; essential in acute cellular knockdown, yet dispensable for adiponectin multimerization in constitutive knockout. The therapeutic corollary is stark: systemic activation or inhibition carries inherent and unpredictable risks, rendering precision strategies mandatory. Recognizing DsbA-L for what it is, a conditional stress adaptor rather than a linear metabolic protector, redirects the field toward mechanism based, context-aware intervention, and away from simplistic agonist or antagonist development.

## Acknowledgments

This work was partially supported by grants 82370858 and 82170886 from the 10.13039/501100001809National Natural Science Foundation of China, 2023JJ10090 from 10.13039/501100019338Distinguished Young Scholar Foundation of Hunan Province.

## Author contributions

Y.D. and L.Y., drafted the manuscript. X.L. and T.X. edited the manuscript. W.M. developed the initial concept and framework for the manuscript and revised the manuscript. All authors contributed to the content, drafting, and critical review of the manuscript.

## Declaration of interests

The authors declare no competing interests.

## Declaration of generative AI and AI-assisted technologies in the writing process

During the preparation of this work, the authors used ChatGPT 5.5 Pro (OpenAI) for language editing and polishing. After using this tool, the authors reviewed and edited the content as needed and take full responsibility for the content of the publication.
